# Novel Automation of an Enzyme-Linked Immunosorbent Spot Assay Testing Method: Comparable Diagnostic Performance of the T-SPOT.*TB* Test Using Manual Density Gradient Cell Isolation versus Automated Positive Selection with the T-Cell *Select* Kit

**DOI:** 10.1128/jcm.00551-22

**Published:** 2022-08-30

**Authors:** Shu-Hua Wang, Murgesean V. S. Rajaram, Andre Trollip, Qian Wu, Doris Ayala, Danyelle Garza, Marielena A. Benavidez, Kelsie Fox, Genesis P. Aguillón-Durán, Erika A. Vargas-Orozco, Lou Torres, Lianbo Yu, S. Rumel Ahmed, Megan Aspden, Thomas Jackson-Soutter, Catherine Coxon, Ruth Brignall, Blanca I. Restrepo

**Affiliations:** a The Ohio State University, College of Medicine, Internal Medicine Department, Infectious Diseases Division, Columbus, Ohio, USA; b The Ohio State University, College of Medicine, Department of Microbiology Infection and Immunity, Columbus, Ohio, USA; c Rapitrade 615 (Pty) Ltd., Somerset West, Cape Town, South Africa; d University of Texas Health Science Center at Houston, School of Public Health in Brownsville, Brownsville, Texas, USA; e Secretaría de Salud de Tamaulipas, Reynosa, Mexico; f NECCR Primacare Research, LLC, Fall River, Massachusetts, USA; g Oxford Immunotec Ltd., Abingdon, Oxford, United Kingdom; h University of Texas Rio Grande Valley, South Texas Diabetes and Obesity Institute, School of Medicine, Edinburg, Texas, USA; University of Manitoba

**Keywords:** ELISPOT, IGRA, T-SPOT.*TB*, tuberculosis, diagnosis

## Abstract

The diagnosis of latent tuberculosis (TB) infection (LTBI) is critical to improve TB treatment and control, and the T-SPOT.*TB* test is a commercial enzyme-linked immunosorbent spot assay used for this purpose. The objective of the study was to increase automation and extend the time between blood collection and processing for the T-SPOT.*TB* test from 0 to 8 h to 0 to 54 h. The previous maximum time between blood collection and processing for the T-SPOT.*TB* test is 32 h using T-Cell *Xtend*. For this, we compared the T-SPOT.*TB* test using manual peripheral blood mononuclear cell (PBMC) isolation by density gradient separation at 0 to 8 h (reference method, control arm) to an automated PBMC isolation method using magnetic beads (T-Cell *Select* kit) at 0 to 55 h postcollection. A total of 620 subjects were enrolled from 4 study sites, and blood samples were collected from each volunteer, comprising 1,850 paired samples in total. Overall agreement between both methods was 96.8% (confidence interval [CI], 95.9 to 97.6%), with 95.8% (CI, 93.5 to 97.5%) positive and 97.1% negative agreement (CI, 96.1 to 97.9%). In summary, there was a strong overall agreement between the automated and manual T-SPOT.*TB* test processing methods. The results suggest that the T-SPOT.*TB* test can be processed using automated positive selection with magnetic beads using T-Cell *Select* to decrease hands-on time. Also, this cell isolation method allowed for the time between blood collection and processing to range from 0 to 55 h. Additional studies in larger and diverse patient populations including immunocompromised and pediatric patients are needed.

## INTRODUCTION

Tuberculosis (TB) affected nearly 10 million people and killed 1.5 million in 2020 (1). Improving diagnosis of latent tuberculosis infection (LTBI) is critical to achieve the World Health Organization (WHO) END-TB targets (2). Approximately one quarter of the world’s population, including more than 13 million people in the United States, have LTBI (3). Globally, the WHO recommends LTBI screening for at-risk populations, including children, people living with HIV, and close contacts of active TB cases, even in high-burden countries (4). The Centers for Disease Control and Prevention (CDC) recommends LTBI screening in individuals at increased risk of reactivation to active TB disease, such as close contacts of newly diagnosed TB patients, individuals with comorbidities, and those planning to start immunosuppressive medications such as tumor necrosis factor alpha (TNF-α) blockers or biologics (5). The U.S. Preventive Services Task Force (USPSTF) recommended screening for LTBI in adults originating from countries with increased TB prevalence regardless of their time in the United States, as well as those from high-risk congregate settings (6). Interferon gamma release assays (IGRAs) offer several advantages over tuberculin skin tests (TST) for LTBI screening (4). Currently, two Food and Drug Administration (FDA)-approved commercial IGRAs are available: QuantiFERON-TB Gold Plus (QFT, Qiagen Ltd.), which utilizes a whole-blood enzyme-linked immunosorbent assay (7), and the T-SPOT.*TB* test (Oxford Immunotec Ltd., Abingdon, UK) a modified enzyme-linked immunosorbent spot (ELISPOT) assay which measures responses at the single-cell level in peripheral blood mononuclear cells (PBMCs) (8). ELISPOT testing platforms are limited by a lack of automation, which compromises the ability of laboratories to scale up testing (9). Increasing automation while allowing the maintenance of flexible times between blood collection and processing would broaden the availability of the T-SPOT.*TB* test, especially in settings with limited laboratory capacity. We aimed to compare the T-SPOT.*TB* test using PBMCs isolated via manual density gradient separation performed between 0 and 8 h after blood collection (reference method, control arm) versus automated positive selection with magnetic beads using the T-Cell *Select* kit performed between 0 and 55 h post-blood collection (experimental) (10).

## MATERIALS AND METHODS

### Study design and participant enrollment.

**FIG 1:**
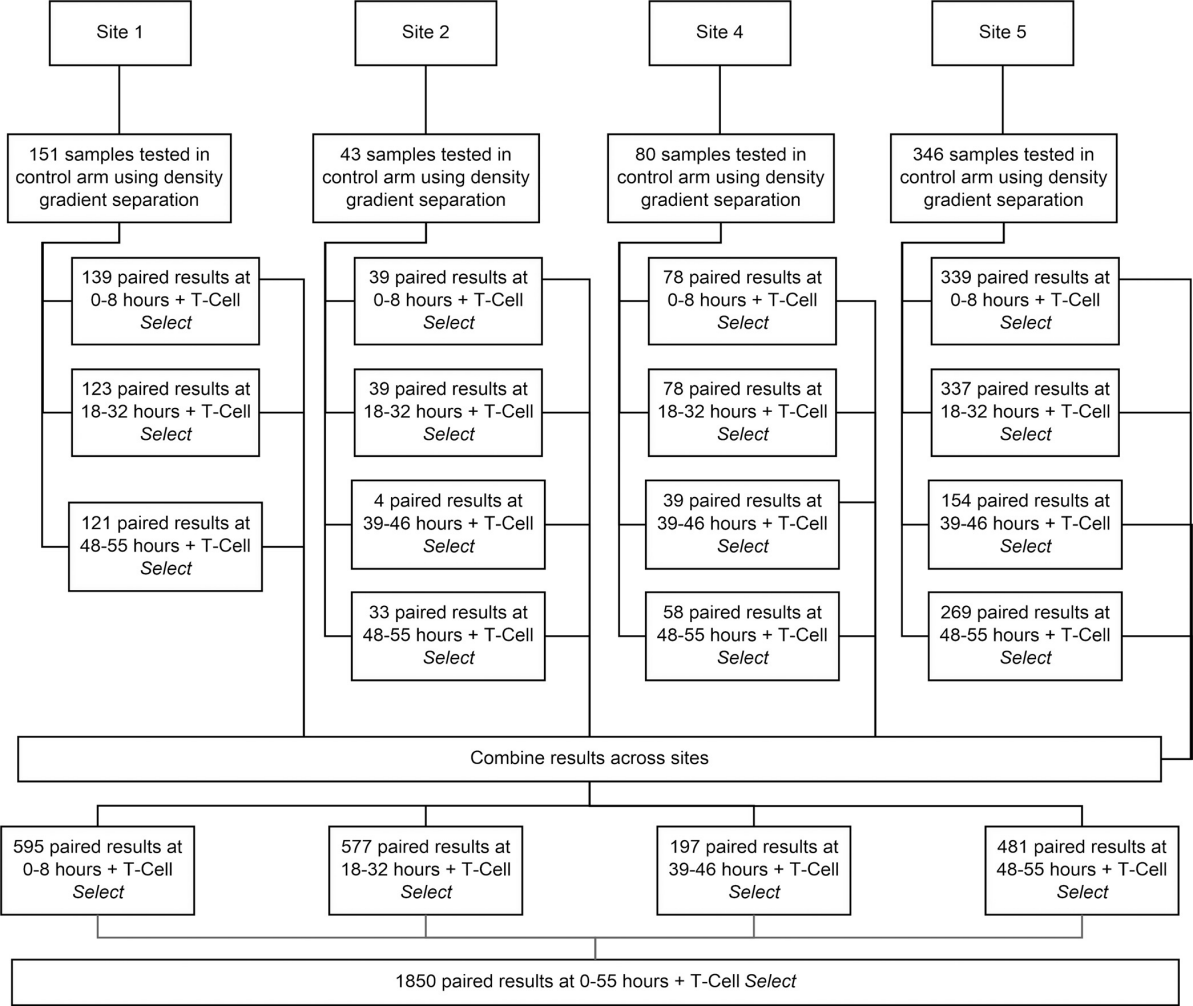
Testing site structure and sample processing.

We conducted a multicenter, concurrent control, matched-pair study, in which test and control specimens were obtained from each study subject during a single blood draw. The study was performed over a 6-month period (between November 2019 and March 2020) at four study sites representing diverse geographic regions and subject populations with various degrees of risk for LTBI or active TB. Site 1: Cape Town, South Africa (high TB endemicity, 24.5% of sample population); site 2: Reynosa, Mexico (Mexico-Texas, USA, border; low to intermediate TB endemicity, 6.9% of the sample population); site 3: Ohio, USA (low to intermediate TB endemicity, 12.9% of the sample population); site 4: Massachusetts, USA (low TB endemicity, 55.8% of sample population). Fig 1 shows the collection and processing scheme for the study. The study size was calculated based on recruitment rates at each site, within the predefined study timeline. However, in total, 616 eligible participants were recruited across all study sites in order to obtain the data required to investigate concordance between the T-SPOT.*TB* methodologies. Participants included adults 18 years or older with either no history of exposure to patients with active pulmonary TB disease (mostly at the low-TB-endemicity site) or having had a close contact with a newly diagnosed TB patient, LTBI, or with present or past TB disease. Classification of LTBI was based on a positive T-SPOT.*TB* test result using the reference test and no TB symptoms, while active TB was based on the presence of signs and symptoms of TB, abnormal chest X ray, or culture confirmation when available. Each study site obtained its own institutional review board (IRB) approval: The Human Sciences Research Council for site 1, the Committee for the Protection of Human Subjects for the University of Texas Health Science Center at Houston and the Ethics Committee from the Secretaría de Salud de Tamaulipas for site 2, the WIRB-Copernicus Group (WCG) for The Ohio State University for site 3, and the Advarra IRB for the New England Center for Clinical Research (NECCR) Primacare Research for site 4. Informed consent was obtained from all study participants.

### T-SPOT.*TB* testing.

Blood samples for each study participant were collected in lithium heparin tubes. The first tube for each study participant was processed within 0 to 8 h from time of collection via density gradient isolation and tested with the T-SPOT.*TB* test as indicated by the manufacturer (no T-Cell *Xtend* reagent added; reference method, control arm) (9). For the experimental arm, the T-SPOT.*TB* tests were performed according to the manufacturer’s instructions between 0 and 55 h (four time blocks of 0 to 8, 18 to 32, 39 to 46, and 48 to 55 h) with cells isolated using the T-Cell *Select* kit (no T-Cell *Xtend* used; a brief description of the T-Cell *Select* kit is detailed in the supplemental Materials and Methods) (11). Laboratory technicians were blinded to enrollment information for removal of bias. Blood remained in the tubes at room temperature, in temperature-controlled boxes (temperature range, 18 to 25°C) until samples were processed. Spot counting was performed independently by each study site using manual counting with a Universal Serial Bus (USB) microscope and confirmed again by an automated plate reader by the sponsor (Cellular Technology Ltd., Ohio, USA), with spot counts compared to capture any discrepancies between methods of reading the count. Any blood samples that contained fewer than 2.0 × 10^6^ viable PBMCs per/mL was not run. This is in accordance with the manufacturer’s instructions that state that the T-SPOT.*TB* test requires 2.5 × 10^5^ viable PBMCs per well, and 4 wells are required for each patient sample. A sample which produced in excess of 10 spots in the nil control, or fewer than 20 spots in the positive control (unless either panel A or panel B is positive, according to a 6-spot cutoff as described below, in which case the result is valid) was considered indeterminate (outside the United States) or invalid (in the Unites States) and is referred to as indeterminate/invalid here (Table 1). The T-SPOT.*TB* test results are interpreted by subtracting the spot count in the nil control well from the spot count in each of the panels. The test result would be classed as positive if panel A minus the nil control is more than or equal to 6 spots. The test result would be considered negative if both panel A minus the nil control) and (panel B minus the nil control are less than or equal to 5 spots, including values less than 0. Where the higher of panel A minus the nil control and panel B minus the nil control is 5, 6, or 7 spots, the result may be considered borderline. Although valid, borderline results are considered less reliable than results where the spot count is farther from the cutoff.

**TABLE 1:** Summary of inconclusive data, including low PBMC recovery, high negative control (Nil) background, and low responsiveness to the positive control (PHA), by control and test arms in all study sites

Site no.	Data [% (*n*)] for:^*a*^					
	Control	0–8 h	18–32 h	39–46 h	48–55 h	Total (combined time points)
Percentage with low PBMC recovery (cell recovery of <2.0 × 10^6^ cells/mL) for time points						
1	2.6 (4/151)	1.3 (2/151)	2.9 (4/139)	NA	3.0 (4/133)	2.4 (14/574)
2	9.3 (4/43)	0 (0/43)	0 (0/39)	0 (0/4)	2.9 (1/34)	3.1 (5/163)
3	0 (0/80)	1.3 (1/80)	1.3 (1/80)	0 (0/40)	1.7 (1/59)	0.9 (3/339)
4	0.6 (2/346)	0 (0/346)	0 (0/341)	0 (0/154)	0.4 (1/273)	0.2 (3/1,460)
All sites	1.6 (10/620)	0.5 (3/620)	0.8 (5/599)	0 (0/198)	1.4 (7/499)	1 (25/2,536)
High nil (>10 spots in nil control well)						
1	2.0 (3/147)	4.1 (6/149)	4.4 (6/135)	NA	3.9 (5/129)	3.6 (20/560)
2	0 (0/39)	0 (0/39)	0 (0/39)	0 (0/4)	0 (0/33)	0 (0/154)
3	0 (0/80)	0 (0/79)	0 (0/79)	0 (0/39)	0 (0/58)	0 (0/335)
4	0.9 (3/344)	0.3 (1/346)	0 (0/341)	0 (0/154)	0 (0/272)	0.3 (4/1,457)
All sites	1.0 (6/610)	1.1 (7/613)	1.0 (6/594)	0 (0/197)	1.0 (5/492)	1.0 (24/2,506)
Low PHA (<20 spots in positive-control well)						
1	0 (0/147)	0 (0/149)	0 (0/135)	NA	0 (0/129)	0 (0/560)
2	0 (0/39)	0 (0/43)	0 (0/39)	0 (0/4)	0 (0/33)	0 (0/158)
4	1.3 (1/80)	0 (0/79)	1.3 (1/79)	0 (0/39)	0 (0/58)	0.6 (2/335)
5	0.3 (1/344)	0 (0/346)	0 (0/341)	0 (0/154)	0.4 (1/272)	0.1 (2/1,457)
All sites	0.3 (2/610)	0 (0/617)	0.2 (1/594)	0 (0/197)	0.2 (1/492)	0.2 (4/2,510)

a NA, not applicable.

### Data analysis.

Data from the study participants, the specimen history, and spot counts were entered into a U.S. FDA 21 Code of Federal Regulations (CFR) Part 11-compliant electronic data capture database (OpenClinica, Massachusetts, USA) and source verified by the sponsor per the International Conference on Harmonization/Good Clinical Practice (ICH/GCP) E6 (R2). All analyses were performed using SAS V9. The analysis comprised data from study participants who had matched experimental versus reference T-SPOT.*TB* test results. Data from all study sites were pooled for the main analysis, and supplementary analyses for individual sites were also performed. The overall agreement between positive and negative specimens was calculated as positive and negative percent agreement with 95% confidence intervals, or kappa statistic, using as a reference the 0- to 8-h time point for density gradient centrifugation. For these calculations, the borderline or indeterminate specimens were excluded given that they would have to be repeated in the clinical setting.

## RESULTS

A total of 620 study participants were enrolled from the four study sites (Fig. 1). Ages ranged from 18 to 85 years; 41% were male; 14% had LTBI and 15% had active TB disease (Table 2 and Table S1 in the supplemental material). Each study participant had specimens processed at different time points, comprising 1,850 paired samples in total. The overall agreement between both methods was 96.8% (CI, 95.9 to 97.6%), with 95.8% (CI, 93.5 to 97.5%) positive and 97.1% negative agreement (CI, 96.1 to 97.9%) (kappa 0.853).

**TABLE 2:** Participant characteristics by study site

Characteristics	Data for:				
	All sites	Site 1	Site 2	Site 3	Site 4
Study site TB endemicity		High	Low to intermediate	Low to intermediate	Low
Study site location		Cape Town, South Africa	Reynosa, Mexico	Ohio, USA	Massachusetts, USA
Sample size (*n*)	620	151	43	80	346
No. HIV positive (%)	42 (7)	39 (25)	0	2 (<1)	1 (<1)
No. with BCG vaccination (%)	317 (51)	91 (60)	37 (86)	16 (20)	173 (50)
Known TB status at the time of enrollment					
No. with no. TB (%)	443 (71)	11 (7)	6 (14)	80 (100)	346 (100)
No. with latent TB infection (%)	87 (14)	51 (34)	36 (84)	0	0
No. with active TB disease (%)	90 (15)	89 (59)	1 (2)	0	0
TB symptoms					
No. with cough >2 wks (%)	101 (16)	95 (63)	2 (5)	1 (1)	3 (<1)
No. with fever (%)	31 (5)	30 (20)	1 (2)	0	0
No. with night sweats (%)	86 (14)	84 (56)	0	0	2 (<1)
No. with wt Loss (>5 kg) (%)	63 (10)	60 (40)	3 (7)	0	0
No. with other (%)	39 (6)	50 (33)	0	0	0
No. with none (%)	498 (80)	38 (25)	39 (91)	79 (99)	342 (99)

Fig 1 shows the number of study participants (*n* = 620) and available paired specimens per study site as follows. For site 1 (Cape Town, South Africa), 151 samples were processed at 0 to 8, 18 to 32, and 48 to 55 h post-blood collection. Site 2 (Mexico) had 43 samples, site 3 (Ohio, USA) had 80 samples, and site 4 (Massachusetts, USA) had 346 samples, tested across all time points. Where fewer than the total number tested at subsequent time points are stated, this is due to low PMBC or negative- or positive-control failure resulting in fewer paired samples being analyzed (Table 1). Furthermore, for purposes of analysis, data from each individual site were combined for time point analysis, resulting in 595 paired samples at 0 to 8 h, 577 paired samples at 18 to 32 h, 197 paired samples at 39 to 46 h, and 481 paired samples at 48 to 55 h post-blood collection. The 39- to 46-h time point was not performed in the high-risk site (site 1) due to logistics. Table 1 illustrates the number of specimens and study sites of origin for low PBMC recovery, negative-control failure (high nil) and positive-control failure (low phytohaemagglutinin [PHA]). Low cell recoveries were higher in sites 1 and 2 than in other study sites, but overall, low cell recovery rates were 1% across all study sites and all time points. Indeterminate/invalid rates due to negative-control failure were slightly elevated in study site 1 compared to the other study sites; however, the negative-control failure rate was not higher than expected overall. Additionally, there is no clear indication of any test arm causing a higher rate of negative-control failures compared to the density gradient control arm. Indeterminate/invalid rates due to positive-control failure were very low across all study sites and all control and test arms. The rate of borderline results was also low across all study sites and control and test arms (Table S1A). In general, higher borderline rates were observed in the test arm; however, this did not impact the clinical concordance as demonstrated in Table 3 and Table S1B.

**TABLE 3:** Summary clinical agreements for 0–55 h for each site

Data analysis set	Overall agreement [% (n; 95% CI)]	Positive agreement with (95% CI)	Negative agreement with (95% CI)
All study results	96.9 (1,792/1,850; 96.0–97.6)	95.9 (397/414; 93.5–97.6)	97.1 (1,395/1,436; 96.1–97.9)
Site 1 results	94.8 (363/383; 92.1–96.8)	98.8 (335/339; 97.0–99.7)	63.6 (28/44; 47.8–77.6)
Site 2 results	88.7 (102/115; 81.4–93.8)	84.9 (45/53; 72.4–93.3)	91.9 (57/62; 82.2–97.3)
Site 3 results	98.0 (248/253; 95.4–99.4)	100 (6/6; 54.1–100.0)	98.0 (242/247; 95.3–99.3)
Site 4 results	98.2 (1,079/1,099; 97.2–98.9)	68.8 (11/16; 41.3–89.0	98.6 (1,068/1,083; 97.7–99.2)
0–8 h (all sites)	96.5 (574/595; 94.7–97.8)	98.0 (145/148; 94.2–99.6)	96.0 (429/447; 93.7–97.6)
18–32 h (all sites)	96.4 (556/577; 94.5–97.7)	94.9 (129/136; 89.7–97.9)	96.8 (427/441; 94.7–98.3)
39–46 h (all sites)	98.0 (193/197; 94.9–99.4)	50.0 (2/4; 6.8–93.2)	99.0 (191/193; 96.3–99.9)
48–55 h (all sites)	97.5 (469/481; 95.7–98.7)	96.0 (121/126; 91.0–98.7)	98.0 (348/355; 96.0–99.2)

The overall positive and negative agreements with 95% CI for each study site are presented in Table 3 (kappa range from 0.731 to 0.921). Overall agreement for all sites was higher than 90%, except for study site 2, which recorded 88.7%. This site recruited fewer study participants overall; therefore, any discordant results have a greater impact on calculated agreements. Positive agreements ranged between 68.8 and 100.0%, with the latter value being obtained in site 4, which was a low-risk TB site, with only 6 positive T-SPOT.*TB* test samples within the data set. The high-risk TB site recorded a positive agreement of 98.8%, and lower agreements in the other two sites are representative of lower-risk status associated with enrollment locations and consequent lower enrollment numbers. Negative agreements ranged between 63.6% and 98.6% between field sites, the latter value being obtained in the low-risk TB site.

With data pooled from all sites, overall positive and negative values between results of the T-SPOT.*TB* test using density gradient and T-Cell *Select* isolation methods exceeded 95%, with the exception of positive agreement at the 39 to 46 h time point, which was not performed at the high-risk TB site. The Cochran-Mantel-Haenzel method provides a pooled estimate of the overall agreement by taking a weighted average, the weight for each study determined by the variability in the agreement estimate for each study. As illustrated in Table 4, overall and negative agreement values when combined in a weighted manner exceeded 95%. Positive agreement values all exceed 95%, with the exception of the 39 to 46 h time point.

**TABLE 4:** Percent agreement using Cochran-Mantel-Haenzel method

Time period (h)	Overall agreement (%) (95% CI)	Positive agreement (%) (95% CI)	Negative agreement (%) (95% CI)
0–8	98.0 (96.6–99.3)	99.1 (96.9–100)	98.4 (96.9–99.9)
18–32	97.2 (95.4–98.9)	97.9 (94.8–100)	97.8 (96.2–99.4)
39–46	99.2 (97.4–100)	50.0 (10.8–100)	99.9 (98.7–100)
48–55	98.9 (97.7–100)	98.8 (96.2–100)	99.9 (99.2–100)
0–55	97.7 (97.0–98.5)	98.6 (97.3–99.9)	98.4 (97.7–99.1)

## DISCUSSION

The T-SPOT.*TB* is a commercial IGRA assay that uses an ELISPOT format for the diagnosis of LTBI. This format requires the separation of PBMCs, which can be an advantage for higher sensitivity when testing individuals with reduced T cell counts, including immunocompromised individuals (11, 12). However, this step requires manual density gradient centrifugations and careful pipetting technique, which limits the automation of the assay. Here, we show for the first time that the magnetic separation of PBMCs using the T-Cell *Select* kit, increases automation of the T-SPOT.*TB* test assay without compromising performance. Testing was conducted in participants from four study sites with diverse population characteristics, including differences in the prevalence of latent TB infection and disease. Furthermore, we found that using the T-Cell *Select* protocol, the time between blood collection and initiation of specimen processing could be delayed by up to 55 h, without addition of the T-Cell *Xtend* reagent. All of this was in contrast to the 0- to 8-h reference standard. During the 55-h period, the blood specimens were kept at room temperature (range, 18 to 25°C), with no need for refrigeration.

A potential concern when testing the T-Cell *Select* kit was the possibility of an increasing proportion of inconclusive results (e.g., low PBMC counts or indeterminate/invalid) due to a hold period for up to 55 h prior to blood processing, combined with the positive selection of cells. However, compared to the control arm, we did not find a significant increase in the proportion of low PBMC recoveries, high background with the nil results, or lack of responsiveness to the positive control with PHA (Table 1). We did observe a higher proportion of low cell recoveries and indeterminates/invalids at site 1 and low cell counts at site 2, which may be inflated by their smaller sample size. The number of indeterminates/invalids due to a failed response to PHA was very low overall at all sites and equated to just 4 samples in the entire data set. Therefore, it can be concluded that cell isolation using the T-Cell *Select* kit, with blood storage of up to 55 h has no significant impact on cell recovery rate or indeterminate/invalid rate. It is worth noting that our study is the first to use an automated platform for a diagnostic assay using an ELISPOT format, and while our approach is described for TB, it can be adapted for use with other pathogens.

As mentioned briefly in the Results section, several sites and time points were observed to have lower overall agreement in results. For example, study site 2 was observed to have a lower overall agreement of 88.7%, but as this site recruited fewer subjects overall, any discordant results had a greater impact on calculated agreements. Further, site 1 recorded a negative agreement of 63.6%, but this low negative agreement value is as expected for an area of high TB endemicity where many individuals may have been exposed to TB unknowingly. Finally, while the positive agreement values overall were in excess of 95%, the 39 to 46 h time point recorded a positive agreement of 50%. However, fewer subjects were enrolled in this cohort (*n* = 4), as site 2 was not designated a high-risk site; therefore, any discordant results had a larger impact on the percent agreement.

We recognize the potential study limitations, largely that this study was focused on the performance of the T-Cell *Select* kit compared to the standard density gradient centrifugation, and hence, only basic clinical information was gathered from the study participants. Therefore, we did not expand the analysis to interpret the data in the context of the clinical findings, including immunocompromised individuals. However, given the high concordance between assays, we do not anticipate any differences in performance between intermediate/high- and low-risk TB study sites. Additionally, this study was designed for analysis when high-, intermediate-, and low-TB risk sites were combined. In future studies it will be important to further investigate the performance of the T-Cell *Select* kit within sites with similar TB risk, particularly those with high endemicity for TB, where there will be high proportions of positive results.

**Conclusions.** There was a strong overall concordance between the automated and manual way of isolating cells for use in the T-SPOT.*TB* test. The T-SPOT.*TB* test can be processed using automated positive selection with T-Cell *Select* to decrease technician hands-on time. T-Cell *Select* allows for blood samples to be stored for 0 to 55 h compared to 32 h in the past, when using T-Cell *Xtend*.
