# Supplementary material for: Novel Automation of an Enzyme-Linked Immunosorbent Spot Assay Testing Method: Comparable Diagnostic Performance of the T-SPOT.TB Test Using Manual Density Gradient Cell Isolation versus Automated Positive Selection with the T-Cell Select Kit
Source: J Clin Microbiol. 2022 Aug 30;60(9):e00551-22. doi: 10.1128/jcm.00551-22 (PMC9491199; doi:10.1128/jcm.00551-22)
Supplement: Supplemental file 1 — Supplemental material. Download jcm.00551-22-s0001.pdf, PDF file, 0.09 MB [file jcm.00551-22-s0001.pdf]

Supplemental material

Methods

The T-Cell *Select* methodology. The T-Cell *Select* kit is intended for the isolation of mononuclear immune cells from whole blood, using positive selection via a magnetic bead-based cell separation system, for use in cell-mediated immune assays.

The use of the T-Cell *Select* kit improves the logistics and workflow of preparing PBMCs for use in ELISPOT assays. The kit contains a proprietary set of reagents consisting of buffer concentrate, antibodies, and superparamagnetic beads. Diluted T-Cell *Select* buffer is added to the whole blood sample to facilitate cell purification and reduce red blood cell contamination, and then antibodies are added which bind to the requisite immune cells in the sample. Addition of superparamagnetic beads results in the formation of complexes with the antibodies attached to the immune cells. The magnetic properties of the beads are utilized, with the aid of a suitable, validated magnetic separation system, to isolate the PBMCs from the sample for subsequent use in the ELISPOT assay.

Results

Supplementary Table 1A: Summary of borderline results for all study sites at all time points

	Borderline (5-7 spots)					
	**Control**	**0-8 Hours**	**18-32 Hours**	**39-46 Hours**	**48-55 Hours**	**Total (combined time points)**
**Site 1**	5.0 % (7/139)	2.2 % (3/139)	5.7 % (7/123)	n/a	1.7 % (2/121)	**3.6 % (19/522)**
**Site 2**	10.3 % (4/39)	10.0 % (4/39)	23.1 % (9/39)	50.0 % (2/4)	3.0 % (1/33)	**13.0 % (20/154)**
**Site 3**	1.3 % (1/78)	2.6 % (2/78)	5.1 % (4/78)	5.1 % (2/39)	3.4 % (2/58)	**3.3 % (11/331)**
**Site 4**	0.3 % (1/339)	4.1 % (14/339)	4.5 % (15/337)	0 % (0/154)	0.4 % (1/269)	**2.2 % (31/1438)**
**Total**	**2.2 % (13/595)**	**3.9 % (23/595)**	**6.1 % (35/577)**	**2.0 % (4/197)**	**1.2 % (6/481)**	**3.3 % (81/2445)**

Supplementary Table 1B: Summary of discordant results as compared to the control arm (0-8 hr density gradient)

			T-SPOT.*TB* + T-Cell *Select* (0-8 hours) (test arm)					Total
			Positive	Borderline			Negative	
			≥8 spots	7 spots	6 spots	5 spots	≤4 spots	
T-SPOT.*TB* (control arm)	Positive		138	1	0	0	1	140
	Borderline	7 spots	2	0	0	0	0	2
		6 spots	4	0	0	0	2	6
		5 spots	1	1	0	0	3	5
	Negative		10	1	5	15	411	442
		TOTAL	155	3	5	15	417	**595**
